# Effects of Nitrogen Application Rate Under Straw Incorporation on Photosynthesis, Productivity and Nitrogen Use Efficiency in Winter Wheat

**DOI:** 10.3389/fpls.2022.862088

**Published:** 2022-03-16

**Authors:** Jinjin Wang, Sadam Hussain, Xu Sun, Peng Zhang, Talha Javed, Eldessoky S. Dessoky, Xiaolong Ren, Xiaoli Chen

**Affiliations:** ^1^College of Agronomy, Northwest A&F University, Yangling, China; ^2^Key Laboratory of Crop Physiology, Ecology and Tillage in Northwest Loess Plateau, Minister of Agriculture, Yangling, China; ^3^Institute of Water Saving Agriculture in Arid Areas of China, Northwest A&F University, Yangling, China; ^4^Department of Agronomy, University of Agriculture Faisalabad, Faisalabad, Pakistan; ^5^College of Agriculture, Fujian Agriculture and Forestry University, Fuzhou, China; ^6^Department of Biology, College of Science, Taif University, Taif, Saudi Arabia

**Keywords:** nitrogen fertilizer, nitrogen use efficiency, photosynthesis, productivity, straw incorporation, winter wheat

## Abstract

Developing a nitrogen fertilizer (N) reduction method under straw incorporation is essentially important for increasing wheat productivity in terms of improved fertilizer use efficiency and high yield in semiarid areas. A two-year field experiment, with five different nitrogen application rates: control (without N application, N0), low N (75 kg ha^–1^, N75), medium N (150 kg ha^–1^, N150), high N (225 kg ha^–1^, N225) and excessive N (300 kg ha^–1^, N300), was conducted in 2018 and 2019 to quantify their impacts on the photosynthetic characteristics, nitrogen utilization (in terms of N accumulation, distribution and transportation, and residual soil NO_3_^–^-N) and productivity of winter wheat. There was a significant impact of N rates on photosynthetic traits, and N accumulation in different organs. As compared with the N300, N150, and N225 improved the photosynthetic characteristics, increased N accumulation in grains by 5.55 and 10.97%, the N contribution proportion of that accumulated after anthesis by 67.90 and 115.56%, and reduced residual N by 62.50 and 46.48%, respectively, thereby effectively improved N absorption efficiency and N contribution rates. Grain yield remained slightly or unchanged among N treatments. Although N0 and N75 treatments reduced the nitrate-N leaching but caused a significant reduction of 18.13 and 28.37%, respectively, in grain yield. From these results, we conclude that N application at 150 and 225 kg⋅ha^–1^ under straw incorporation was the most effective fertilization method in achieving the higher photosynthetic characteristics, improving NUE and grain yield. This study provides theoretical and practical guidance for wheat production techniques.

## Introduction

Fertilizers, when applied at appropriate rate, have played an important role in increasing the crop productivity (Morari et al., 2011). Good fertilization practices, including as per recommended methods and rates, are very important not only for enhancing crop productivity but ensuring soil and environmental health (Yousaf et al., 2016a,b; Shah et al., 2017a). However, inadequate application of synthetic fertilizers, particularly nitrogenous fertilizers, have a negative impact on crop growth, the soil and the environment; it is predicted that increasing N fertilizers may cause an increase of 23–60% in N_2_O emissions by 2030 (FAO, 2009). China is among the largest consumer of chemical fertilizers in the world and overuse of synthetic fertilizers, including nitrogenous fertilizers, is a common practice to achieve the higher crops yield (Du et al., 2020). Increasing N inputs would decrease nitrogen use efficiency (NUE) and increase N losses and environmental pollution (Shah et al., 2017b; Bhattacharya, 2019; Zhang et al., 2019; Bhattarai et al., 2021). In a recent study, Gu et al. (2019) also demonstrated that long-term use of nitrogenous fertilizers has been resulted in enhancing the acidification, degradation, and soil compaction problems. Agricultural management practices, for example, the application of N fertilizers greatly influence soil health in term of soil organic carbon (SOC) content, crop growth, and developmental processes. Various reports have reported that reducing mineral N application along with the application of manures would be one of the best practices for enhancing crop performance through promoting soil health and NUE (Duan et al., 2016).

Guanzhong Plain, having an area of ∼ 34000 km^2^, is an important grain production base in Shaanxi Province and in China (Liu et al., 2014). Winter wheat is the main grain crop in this region. This region is rich in straw resources, but at the same time, the phenomenon of straw burning is very common (Zhu et al., 2016; Sun et al., 2017). Intensive planting mode without straw retention/incorporation reduced the soil organic matter and soil nutrients which reduced the reward effect of chemical fertilizer (Scotti et al., 2015). In order to pursue high crop yield and reduce cost, the phenomenon of excessive application of N and the “one blast” fertilization method are prevalent (Lou et al., 2011; Ju and Gu, 2014; Zhao et al., 2016; Hafeez et al., 2019; Shah et al., 2021a,b; Yousaf et al., 2021), causing serious loss of N and reduced NUE, which not only wastes resources but also causes serious environmental pollution (Jin et al., 2012; Wei et al., 2015; Zhao and Yin, 2015). Agricultural production in this region lacks scientific and reasonable fertilization guidance.

Straw incorporation can increase soil organic matter, reduce fertilizer input, and increase the reward effect of chemical fertilizers (Zhang et al., 2016). It is commonly considered that fertilizers play a significant role in the continuous increase in the productivity of agricultural crops (Lou et al., 2011). Currently, considering the environmental benefits and better crop yield that sustainable agriculture management provides, a combination of N fertilizer application with straw incorporation is recommended as a sustainable and environment-friendly approach by researchers (Bakht et al., 2009; Liao et al., 2015; Xu et al., 2019; Zhang et al., 2019) and government agencies (MEP, 1999; MOA-PRC, 2015) to increase the soil EC, improve soil fertility status and minimize the environmental problems. Crop straw incorporation or retention is commonly believed to have a positive influence on the soil quality, soil carbon and N dynamics (Ghimire and Machado, 2015). It will promote soil productivity, soil organic C sequestration and NUE (Liu et al., 2014). Various studies have demonstrated that straw incorporation can enhance the carbon inputs which can result in enhancing the organic matter contents and accumulation of essential nutrients that ultimately enhance nutrient use efficiency of crop plants (Li et al., 2010; Tong et al., 2014; Yuan et al., 2021). It is also well documented that straw incorporation positively affected the activities of various beneficial microbes to facilitate in protecting the soil environment for sustainable agricultural production (Garg and Bahl, 2008). However, results of previously published reports on the efficacy of straw incorporation treatments for crop yield and environment were inconsistent, showing either positive (Huang et al., 2017), negative (Yao et al., 2017; Xu et al., 2021), or neutral effects (Zhang et al., 2017). Fertilization had a significant effect on soil health and productivity, and soil GHGs emissions (Liu et al., 2017). N application, when exceeds crop demand, has been reported to increase the primary production of GHGs, particularly N_2_O, in most terrestrial ecosystems, mainly due to its primary effect on soil N pool (Elser et al., 2007; Xu et al., 2019) and decreasing the NUE (Zhang et al., 2019). Therefore, it is imperative to find the best application rate to reduce the N losses and associated environmental problems.

At present, only limited studies have investigated the effects of reducing N on the basis of straw incorporation for winter wheat production in semiarid areas (Weller et al., 2015; Liang et al., 2017). And the conclusion is still inconclusive (Yin et al., 2018). Furthermore, there is still a lack of knowledge whether better photosynthetic processes, crop productivity and high NUE can be reconciled by optimizing fertilization regimes under straw incorporation. Thus, in this study, in order to find the best fertilization management, we set decreasing N fertilization treatments under the condition of traditional N to find the best choice for increasing the productivity of the wheat crops. Specific objectives of this study were to: (1) investigate the effect of N fertilizer on wheat growth, photosynthetic characteristics, N utilization (N accumulation, distribution and transportation, and residual soil –_3_^–^-N) and grain yield; (2) and provide a new direction for optimizing wheat N fertilizer management in semi-arid areas of China. For this study, we hypothesized that the conventional rate of N replaced by reduced N dose would promote the photosynthetic process, crop productivity, increase NUE in terms of high N accumulation in different plant organs and reduce leaching losses under straw incorporation conditions. Results of this experiment would help to identify the most efficient N application rate for achieving better crop growth, sustainable wheat grain yield, and high NUE under straw incorporation in semiarid areas.

## Materials and Methods

### Experimental Site Description

**FIGURE 1:**
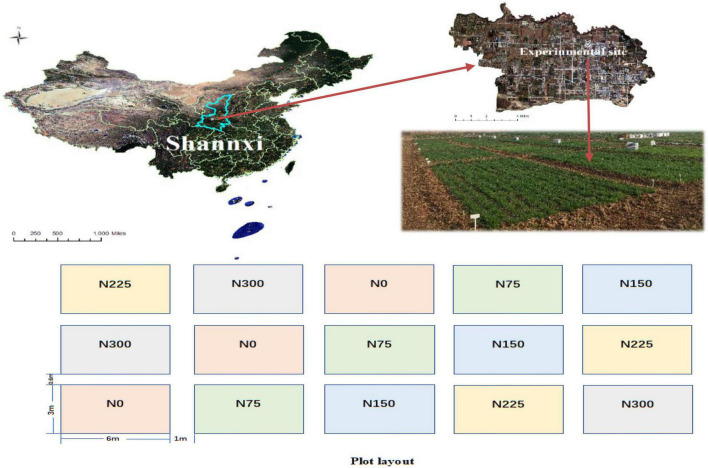
Location of the experimental site and layout of the field plots randomly distributed with N application rates. N0, unfertilized control; N75, 75 kg N⋅ha^– 1^; N150, 150 kg N⋅ha^– 1^; N225, 225 kg N⋅ha^– 1^; N300, a traditional N application rate 300 kg N⋅ha^– 1^.

Field experiments were conducted over two consecutive years (2018–2019) at the Institute of Water-saving Agriculture in Arid Areas of China (IWSA), at Northwest A&F University, Yangling (E108°04′, N35°20′) Shaanxi Province, China (Figure 1). This area is typically a semi-arid area which is located at the northern foot of the Qinling Mountains and on the Toudaoyuan in the west of the Weihe Plain. The experimental site is characterized as a hilly, dry flat land in the abdomen of the Loess Plateau with Lou soil type. Table 1 shows the nutrient content of wheat straw for returning to the field during the next wheat season and the soil nutrient content of the 0–60 cm soil layer before planting. Soil physical and chemical properties of above 0–20 cm were as follows: (i) soil bulk density 1.25 ± 0.3 g⋅cm^–3^, SOM 11.97 ± 0.95 g⋅kg^–1^, available N 53.12 ± 2.45 mg⋅kg^–1^, available P 22.34 ± 1.24 mg⋅kg^–1^, available K 97.37 ± 4.56 mg⋅kg^–1^ and pH 7.59 ± 0.58.

**TABLE 1:** Nutrient contents of incorporated wheat straw and soil nutrient contents in different soil layers before planting (g⋅kg^–1^).

Nutrient	Soil layer (cm)			Crop straw (g⋅kg^–1^)
	
	0–20	20–40	40–60	
Organic C (g⋅kg^–1^)	11.97	12.03	8.25	/
Total N (g⋅kg^–1^)	1.31	0.78	0.56	6.64
Total P (g⋅kg^–1^)	0.729	0.54	0.39	0.47
Total K (g⋅kg^–1^)	14.9	20.14	25.48	20.62

**FIGURE 2:**
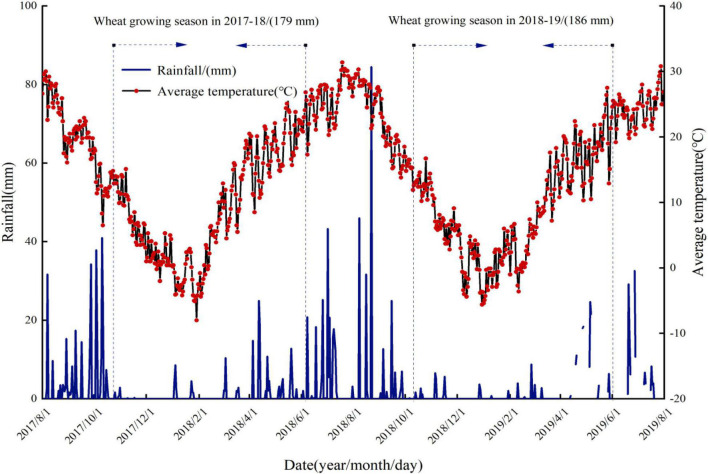
Rainfall and average temperature during the winter wheat growing seasons in 2018 and 2019 in Yangling, China.

In the past 40 years, the average annual temperature is 13.5°C, annual average rainfall was 540–625 mm, where most of rainfall occurred during July and September, and annual evaporation was 993.2 mm. The average temperature and rainfall during the growth period of wheat are shown in Figure 2. The average temperature during the whole growth period of winter wheat (from October to June) in the first year (2017–2018) and second year (2018–2019) of the experiment was 9.0 and 8.9°C, respectively. The rainfall during the whole growth period in the first year (2017–2018) and second year (2018–2019) of the experiment was 179.0 and 186.0 mm, respectively.

### Experimental Design and Treatments

The area planting system is winter wheat - leisure. The seedbed preparation included mechanical crushing of the whole wheat straw and return to the soil, this was followed by 15 cm deep plowing. The wheat straw (4500 kg⋅ha^–1^) was returned to the field before sowing. The nutrient contents of used straw were: (i) 6.64 ± 0.56 g kg^–1^ total N, (ii) 0.47 ± 0.01 g kg^–1^ total phosphorus (P), (iii) 20.62 ± 1.21 g kg^–1^ total potassium (K). The site was planted with winter wheat, where its cultivar Xinong-979 was sown, keeping an inter-row spacing of 20 cm. The seeds of winter wheat were sown in the mid-October and harvested in the first week of June during both study years. The plot area was 18 m^2^ (6 m × 3 m), with 3 replicates and a completely random arrangement. No irrigation was applied during the growing period of crop and other management practices were the same as local practices. The recommended dose of phosphorus and potassium was applied at 150 kg⋅ha^–1^ P_2_O_5_ and 60 kg⋅ha^–1^ K_2_O, using calcium phosphate and potassium chloride. N fertilizer using urea, five N treatments (0, 75, 150, 225, and 300 kg⋅ha^–1^, abbreviated as N0, N75, N150, N225, N300), was applied as the basal application to be consistent with the local farmer community.

### Sampling Methods and Measurements

#### Plant Sampling

##### Leaf Area Index and Photosynthetic Indices

In the crucial growth period of winter wheat, at the flowering stage, values were recorded during a sunny day between 9:00 – 11:00 a.m. The net photosynthetic rate (Pn), stomatal conductance (Gs), transpiration rate (Tr) and intercellular CO_2_ concentration (Ci) of flag leaves were measured by li-6400 photosynthetic apparatus manufactured in the United States, and the SPAD values using flag leaves were measured by SPAD apparatus, which was repeated for 10 times. The leaf area per plant was measured at the jointing stage, flowering stage, middle and late filling stage. Ten plants having similar growth trends were selected for each experimental plot, and the length and width of leaves were measured from all green fully expanded leaves. Then, the length and width coefficient method was applied to calculate the leaf area of each leaf by using the following equation: leaf area = length × width × 0.75. Leaf area index (LAI) was calculated as follows:


(1) $$\text{LAI}=\frac{\mathrm{Total}\,\mathrm{leaf}\,\mathrm{area}\,\left({\text{m}}^{2}\right)}{\mathrm{Total}\,\mathrm{area}\,\mathrm{of}\,\mathrm{cultivated}\,\mathrm{land}\,\left({\text{m}}^{2}\right)}$$

##### Indicators Related to Nitrogen Accumulation and Transport

At the flowering and maturity stage, samples were taken from the center of the experimental plot dedicated to sampling, and ten (10) plants were sampled from each plot, and further separated into different parts as: leaf, stem + leaf sheath, glume + cob, grain and other organs. For analyzing the N concentration through the Kjeldahl method, firstly, the samples were oven-dried at 110 °C for 30 min, and later at 80°C to get a constant weight. Nitrogen accumulation and translocation in plant organs were calculated according to the method of Stevens et al. (2005) as follows:


(2) $$\mathrm{Nitrogen}\,\mathrm{accumulation}\,\mathrm{in}\,\mathrm{various}\,\mathrm{organs}\,\left(\text{kg}\cdot {\text{ha}}^{-1}\right)=\mathrm{Nitrogen}\,\mathrm{content}\times \mathrm{Dry}\,\mathrm{matter}\,\mathrm{mass}$$

(3) $$\mathrm{Nitrogen}\mathrm{distribution}\mathrm{ratio}\mathrm{of}\mathrm{each}\mathrm{organ}\left(\%\right)=\frac{\mathrm{Nitrogen}\,\mathrm{accumulation}\,\mathrm{of}\,\mathrm{each}\,\mathrm{organ}}{\mathrm{Nitrogen}\,\mathrm{accumulation}\,\mathrm{of}\,\mathrm{single}\,\mathrm{plant}}\times 100$$

(4) $$\mathrm{Nitrogen}\,\mathrm{translocation}\,\mathrm{in}\,\mathrm{vegetative}\,\mathrm{organs}\,\left(\text{kg}\cdot {\text{ha}}^{-1}\right)={\text{NAVO}}_{\text{F}}-{\text{NAVO}}_{\text{M}}$$

Where, NAVO_*F*_ and NAVO_*M*_ are the accumulation of nitrogen in vegetative plant organs at flowering and maturity stage, respectively (kg⋅ha^–1^).


(5) $$\mathrm{Nitrogen}\,\mathrm{transport}\,\mathrm{rate}\,\mathrm{of}\,\mathrm{vegetative}\,\mathrm{organs}\%=\frac{\mathrm{Nitrogen}\,\mathrm{transport}\,\mathrm{volume}\,\mathrm{of}\,\mathrm{vegetative}\,\mathrm{organs}}{{\text{NAVO}}_{\text{F}}}$$

#### Soil Sampling and Measurements

Soil samples were collected from three random soles from a 2 m soil layer from the center of each plot after harvesting winter wheat in early June of 2018 and 2019. Three sets of soil samples from each experimental unit were randomly taken from three locations within the 0–200 cm soil profile by using a core sampler (15 cm high and 5 cm diameter). Well sieved soil samples (2 mm), from deep 2 m soil layer, were refrigerated to measure the soil NO_3_^–^-N concentration for consideration of NH_3_^–^-N leaching. A mixture containing 5.0 g of fresh sieved soil (<2 mm) extracted with 50 ml of 1 mol⋅L^–1^ KCL solution was used for determining the soil total NH_4_^+^-N and NO_3_^–^-N. After filtration with Whatman filter paper (No. 42), AA3 continuous flow analyzer was used to analyze the extract solution, according to the method of Norman and Stucki (1981). The soil bulk density was determined by the ring knife method, for that, five replicates were taken from the top-soil layer and three replicates were from below the soil layer. The samples were put in an oven at 105 ± 2 °C for 4 h, and then cooled the samples in a desiccator and weigh to a constant weight.

The nitrogen harvest index (NHI), agronomic efficiency nitrogen (AEN), partial factor productivity (PFP) and recovery efficiency nitrogen (REN) were used for the determination of nitrogen use efficiency (NUE), according to the procedure of (Dobermann, 2005; Jin et al., 2012; Hartmann et al., 2015) as follows:


(6) NHI=GN/TPN

(7) $$\text{AEN}=\frac{\text{Y–Y}\,0}{\text{F}}$$

(8) PFP=Y/F

(9) $$\text{REN}=\frac{\mathrm{TPN}-\mathrm{TP0}}{\text{F}}$$

Where GN is nitrogen content in grains; TPN denotes the total uptake of N by the wheat plant; TP0 denotes the total uptake of nitrogen by the wheat plant under control condition; F denotes the amount of applied N; Y is the grain yield with applied N; Y0 is the crop yield under N0 treatment.

**NO_3_** ^–^ **-N content was calculated as follows:**


(10) $$c=\frac{c_{0}\times v_{T}\times 50\times 1000}{v\times w\times 1000}$$

Where c denotes the quantity of soil NO_3_^–^ -N (mg⋅kg^–1^); C_0_ is the mass concentration of test solution obtained from the curve (μg⋅mL^–1^); 50 is the total volume of the colorimetric test solution used (mL); v_*T*_ denotes the total extract volume used (mL); v is the volume of the filtrate drawn; w is the mass of the dry soil sample (g).

The accumulation of NO_3_^–^-N in the soil is the sum of the individual accumulated amount of NO_3_^–^-N in each soil layer at a depth of 0–2 m, the formula is as follows:


(11) $$\text{A}=\text{c}\times \text{h}\times \text{BD}\times \frac{10}{100}$$

Where A and c are the accumulation of NO_3_^–^-N (kg⋅ha^–1^) and soil layer NO_3_^–^-N concentration (mg⋅kg^–1^), respectively; BD and h are the soil bulk density (g⋅cm^–3^) and thickness of soil layer (cm), respectively.

Residual nitrogen was calculated as follows:


(12) $$\mathrm{Fertilizer}\,\mathrm{nitrogen}\,\mathrm{residue}={\text{A}}_{\text{n}}-\text{A}$$

Where A_*n*_ and A are the accumulation of NO_3_^–^-N in fertilized area after planting the crop (kg⋅ha^–1^) and the accumulation of NO_3_^–^-N in the non-fertilized area (kg⋅ha^–1^), respectively.


(13) $$\mathrm{Fertilizer}\mathrm{nitrogen}\mathrm{apparent}\mathrm{residue}\mathrm{rate}\left(\%\right)=\frac{{\text{A}}_{\text{n}}-A}{\text{c}}$$

Where c is the total N application (kg⋅ha^–1^).

#### Grain Yield

At maturity, three central rows were harvested from each plot. The harvested plants were threshed using a mini thresher to separate the grains. The grains were weighed to record the grain yield.

##### Statistical Analysis

Primary data from the experimental unit were computed using Microsoft Excel 2010. Differences among the nitrogen treatments were separated by analysis of variance technique, and mean values were compared to calculate the least significant difference (LSD) at the 0.05 level using SPSS-19.0 software. Origin 2021 software was used for regression analysis and graphing.

## Results

### Photosynthetic Indicators

**FIGURE 3:**
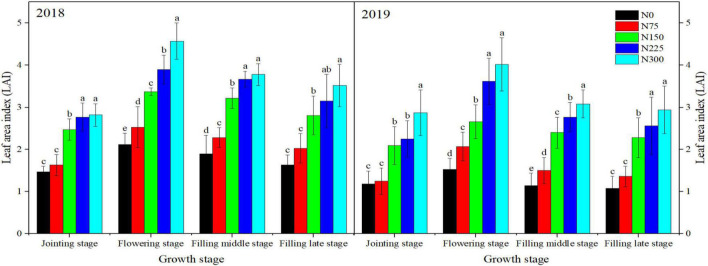
Impact of different nitrogen (N) application rates on leaf area index at different growth stages in winter wheat during 2018 and 2019. Above bars, different small letters show significant differences among N treatments at each growth stage. N0: unfertilized control; N75: N application at 75 kg N⋅ha^– 1^; N150: N application at 150 kg N⋅ha^– 1^; N225: N application at 225 kg N⋅ha^– 1^; N300: traditional N application rate of 300 kg N⋅ha^– 1^.

**FIGURE 4:**
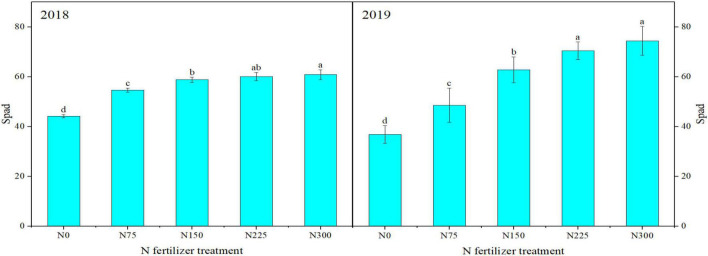
Influence of different nitrogen (N) application rates on SPAD values at flowering stage in winter wheat during 2018 and 2019. Above bars, different small letters show significant differences among N treatments. N0: unfertilized control; N75: N application at 75 kg N⋅ha^– 1^; N150: N application at 150 kg N⋅ha^– 1^; N225: N application at 225 kg N⋅ha^– 1^; N300: traditional N application rate of 300 kg N⋅ha^– 1^.

Nitrogen treatments significantly affected the photosynthetic traits during both study years. As sown in Figures 3, 4, maximum LAI at different growth stages and SPAD values were recorded for N300 which were statistically the same as N225 during both study years. However, minimum values of these traits were recorded for N0 followed by N75, N150, and N225 (Figures 3, 4). Similarly, higher net photosynthetic rate (Pn), stomatal conductance (Gs), and transpiration rates were recorded for N225 during both study years as compared with N0. When compared with control, N225 increased Pn by 113.12 and 25.43%, Gs by 188.89 and 25% and transpiration rate by 155.92 and 17.39% in 2018 and 2019, respectively (Table 2). In contrast, N225 treatment recorded a maximum reduction in intercellular CO_2_ concentration by 27.08 and 9.03% in 2018 and 2019, respectively, compared to the control treatment.

**TABLE 2:** Effects of different N application rates on net photosynthetic rate, stomatal conductance, intercellular CO_2_ concentration and transpiration rate at flowering stage of winter wheat in 2018 and 2019.

Year	Treatments	Index			
		
		Net photosynthetic rate	Stomatal conductance	Intercellular CO_2_ concentration	Transpiration rate
		
		(μmol m^–2^ s^–1^)	(mmol m^–2^⋅s^–1^)	(μmol mol^–1^)	(mmol m^–2^ s^–1^)
2018	N0	12.35e	0.09d	180.98a	2.70e
	N75	13.91d	0.09d	163.38b	2.84d
	N150	18.98c	0.16c	140.62e	4.36c
	N225	26.32a	0.26a	131.96d	6.91a
	N300	22.62b	0.22b	152.31c	6.18b
2019	N0	8.81c	0.32b	362.10a	1.61b
	N75	10.16b	0.33b	348.82b	1.70b
	N150	10.89a	0.37ab	338.60c	1.78ab
	N225	11.05a	0.40a	332.85c	1.89a
	N300	10.62ab	0.34b	350.94b	1.69b

*N0: unfertilized control; N75: N application at 75 kg N⋅ha^–1^; N150: N application at 150 kg N⋅ha^–1^; N225: N application at 225 kg N⋅ha^–1^; N300: traditional N application rate of 300 kg N⋅ha^–1^. Different small letters show significant differences at p < 0.05.*

### Distribution of Nitrogen

Nitrogen distribution in different plant organs was significantly affected by N treatments during both experimental years at the flowering and maturity stage (Tables 3, 4). At the flowering stage, maximum N accumulation in spike axis + husk and leaf were recorded for N300 which was statistically the same as N225, however, maximum N accumulation in stem + sheath was recorded for N225 during both years. For total N accumulation, maximum values were recorded for N330 during both years, which was statistically similar to N225 in 2018. At maturity stage, maximum N accumulation in grains, spike axis + husk, and leaf + spike + sheath was recorded for N300 followed by N225 during both experimental years (Table 4). Similarly, for total N accumulation, maximum values were recorded for N300 which were statistically similar to N225. For total distribution proportion, maximum values (18.40 and 16.88%, in 2018 and 2019, respectively) were recorded for N300 flowered by other N treatments.

**TABLE 3:** Nitrogen accumulation and distribution proportion in different organs at flowering stage of winter wheat in 2018 and 2019 under different nitrogen application rates.

Year	N treatments	Nitrogen accumulation amount (kg⋅hm^–2^)				Distribution proportion (%)		
			
		Spike axis+Husk	Leaf	Stem+Sheath	Total	Spike axis+Husk	Leaf	Stem+Sheath
2018	N0	11.23c	20.02c	20.69b	51.94d	21.57a	38.46b	39.97a
	N75	15.64c	30.73c	26.41b	72.78c	21.67a	42.17ab	36.16ab
	N150	22.63b	45.26b	33.62ab	101.51b	22.42a	44.55a	33.03ab
	N225	29.65a	56.34ab	38.56a	124.55a	24.15a	44.99a	30.86b
	N300	32.85a	60.35a	42.16c	135.36a	24.15a	44.44a	31.41ab
2019	N0	15.62c	22.16e	21.56b	59.34e	26.27a	37.52b	36.21a
	N75	17.92bc	32.85d	26.39b	77.16d	23.27ab	42.58ab	34.15a
	N150	22.03b	43.33c	31.48ab	96.84c	22.78ab	44.75a	32.47a
	N225	26.54ab	56.32b	36.85a	119.71b	22.13ab	47.16a	30.71a
	N300	29.53a	65.12a	41.59c	136.24a	21.61b	47.81a	30.58a

*N0: unfertilized control; N75: N application at 75 kg N⋅ha^–1^; N150: N application at 150 kg N⋅ha^–1^; N225: N application at 225 kg N⋅ha^–1^; N300: traditional N application rate of 300 kg N⋅ha^–1^. Different small letters show significant differences at p < 0.05.*

**TABLE 4:** Nitrogen accumulation and distribution proportion in different organs at maturity stage of winter wheat in 2018 and 2019 under different nitrogen application rates.

Year	Treatment	Nitrogen accumulation amount (kg⋅hm^–2^)				Distribution proportion			
			
		Grain	Plant organs			Grain	Plant organs		
					
			Spike axis+Husk	Leaf+Stem+Sheath	Total		Spike axis+Husk	Leaf+Stem+Sheath	Total
2018	N0	66.14c	3.24b	4.34c	7.58d	89.72a	3.49d	6.10b	10.28b
	N75	87.40c	4.47b	6.35c	10.82cd	88.98a	4.78cd	6.78b	11.02b
	N150	120.56b	6.35b	10.15bc	16.50c	87.95a	6.73c	7.80b	12.05b
	N225	173.56a	10.56a	14.26b	24.82b	97.39a	10.76b	7.63b	12.61b
	N300	169.87a	13.59a	25.00a	38.59a	81.60b	14.27a	12.77a	18.40a
2019	N0	86.24d	3.74d	8.85c	12.59e	87.27a	4.11d	9.30a	12.73b
	N75	110.26c	5.10cd	12.06c	17.16d	86.40ab	5.58cd	9.98a	13.60b
	N150	135.86b	6.37c	15.96b	22.33c	85.87ab	6.90c	10.53a	14.13b
	N225	170.65a	8.56b	20.67a	29.23b	85.28ab	9.13b	10.89a	14.72b
	N300	168.52a	11.51a	22.63a	34.14a	83.12b	12.15a	11.87a	16.88a

*N0: unfertilized control; N75: N application at 75 kg N⋅ha^–1^; N150: N application at 150 kg N⋅ha^–1^; N225: N application at 225 kg N⋅ha^–1^; N300: traditional N application rate of 300 kg N⋅ha^–1^. Different small letters show significant differences at p < 0.05.*

### Transport of Nitrogen

**FIGURE 5:**
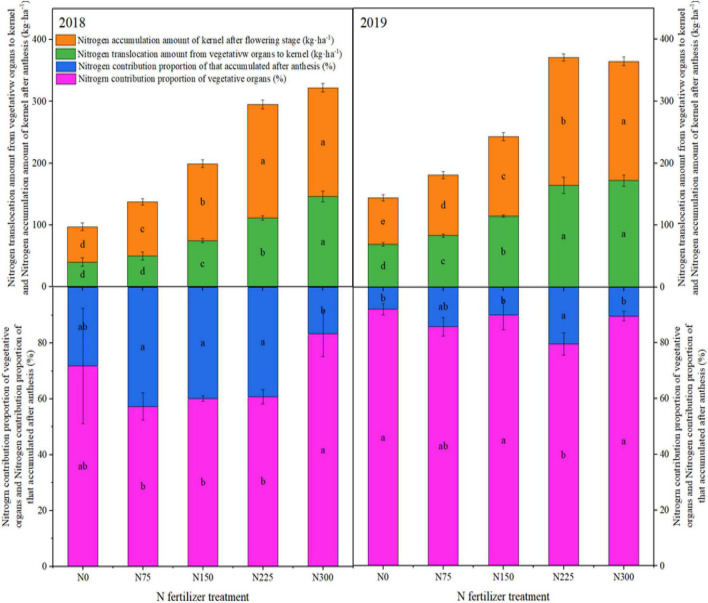
Influence of different nitrogen (N) application rates on N accumulation amount of kernel after flowering stage, N translocation amount from vegetative organs to the kernel, N contribution proportion of that accumulated after anthesis stage and N contribution proportion from vegetative organs in winter wheat during 2018 and 2019. In bars of specific colors, different small letters show significant differences among N treatments. N0: unfertilized control; N75: N application at 75 kg N⋅ha^– 1^; N150: N application at 150 kg N⋅ha^– 1^; N225: N application at 225 kg N⋅ha^– 1^; N300: traditional N application rate of 300 kg N⋅ha^– 1^.

Nitrogen accumulation amount in kernels after flowering stage, N translocation amount from vegetative organs to kernel, N contribution proportion of that accumulated after anthesis stage and N contribution proportion from vegetative organs varied significantly for N treatments where maximum N accumulation amount in kernels after flowering stage and N translocation amount from vegetative organs to kernels was recorded for N300 followed by N225 whereas minimum was recorded for control treatment without N application during both study years (Figure 5). For N contribution proportion of that accumulated after anthesis stage, maximum values were recorded for N75, N150, and N225 followed by control treatment without N application, however, minimum was recorded for N300. Similarly, for N contribution proportion from vegetative organs, N300 and N0 significantly increased the values followed by other treatments (Figure 5).

### Nitrogen Efficiency

As shown in Table 4, NHI was decreased with increasing N rates during both study years. However, for N fertilizer recovery efficiency, values were increased with increasing N rate in 2018, and the values were statistically the same for all N treatments in 2019. For N fertilizer contribution rate, the maximum percentage of 22.69 and 40.02% in 2018 and 2019 were recorded for N225 as compared with N0 where minimum values were recorded (Table 5). However, there was an opposite trend for N-fertilizer partial factor productivity and agronomic efficiency of N fertilizer where maximum values were recorded for control treatment without N application.

**TABLE 5:** Effect of nitrogen (N) application rates on grain yield, N fertilizer recovery, N fertilizer contribution efficiency, N fertilizer partial factor productivity and agronomic efficiency of N fertilizer in winter wheat during 2018 and 2019.

Year	Treatment	Grain yield (kg⋅hm^−^^2^)	N harvest index	N-fertilizer recovery efficiency (kg kg^–1^)	Nitrogen fertilizer contribution rate (%)	N-fertilizer partial factor productivity (kg kg^–1^)	Agronomic efficiency of N-fertilizer (kg kg^–1^)
2018	N0	5186.61d	0.897a	/	/	/	/
	N75	6006.38c	0.890ab	32.67b	15.05c	80.09a	12.50a
	N150	6490.00b	0.880ab	42.23ab	21.26b	43.02b	9.18b
	N225	6600.00a	0.874b	55.41a	22.69a	29.33c	6.66bc
	N300	6550.00b	0.816c	44.91a	21.62b	21.70d	4.69c
2019	N0	4104.16d	0.873a	/	/	/	/
	N75	5254.69c	0.864ab	38.12a	21.88c	70.06a	15.34a
	N150	6500.86b	0.859ab	39.57a	36.82ab	43.34b	15.98a
	N225	6845.47a	0.853ab	44.91a	40.02a	30.42c	12.18b
	N300	6405.68b	0.831b	34.61a	35.88b	21.35d	7.67c

*N0: unfertilized control; N75: N application at 75 kg N⋅ha^–1^; N150: N application at 150 kg N⋅ha^–1^; N225: N application at 225 kg N⋅ha^–1^; N300: traditional N application rate of 300 kg N⋅ha^–1^. Different small letters show significant differences at p < 0.05.*

### Residual Nitrogen

**FIGURE 6:**
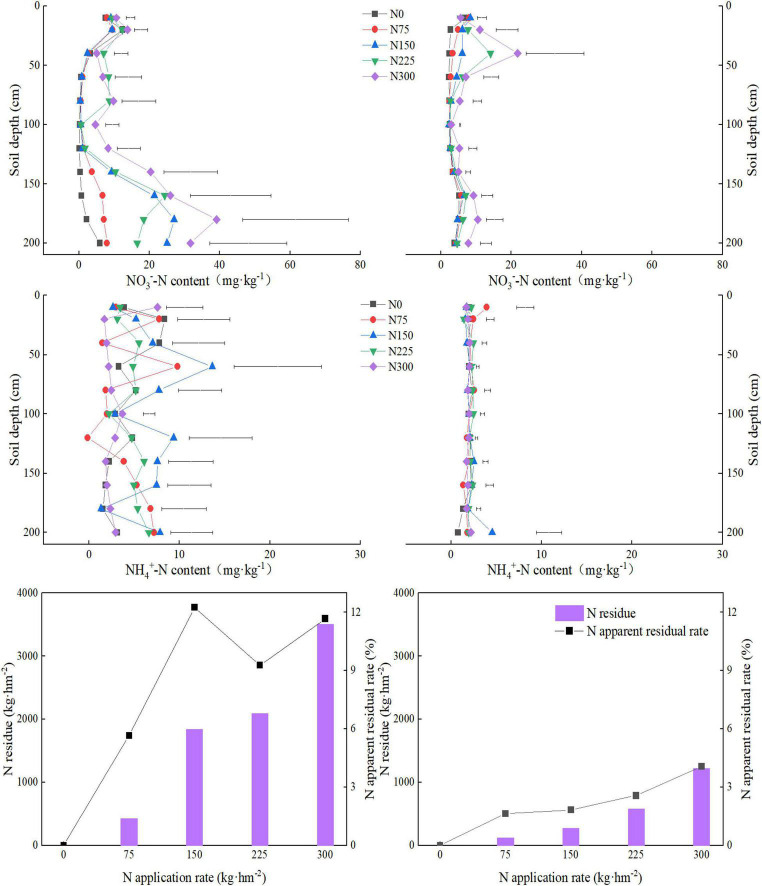
Effect of different nitrogen (N) fertilization rates on NH_3_^–^-N, NH_4_^+^-N, residual N and N apparent residual rate in winter wheat during 2018 and 2019. The line segment is the LSD-t value obtained by multiple comparisons using the LSD-t method. N0: unfertilized control; N75: N application at 75 kg N⋅ha^– 1^; N150: N application at 150 kg N⋅ha^– 1^; N225: N application at 225 kg N⋅ha^– 1^; N300: traditional N application rate of 300 kg N⋅ha^– 1^. The left-side figures show data for 2018, and the right-side figures show data for 2019.

The NH_3_^–^-N, NH_4_^+^-N, residual N and N apparent residual rates in 2018 and 2019 are shown in Figure 1. The NH_3_^–^-N contents increased with the increase in soil depth and N application rates (Figure 6). The straw effect gradually appeared over time, which reduced the NH_3_^–^-N leaching during the second year. The positive influence of N treatments on NH_4_^+^-N was as N150 > N225 > N75 > N0 > N300. Residual N amount and rates were increased gradually with the increase of N application rate in 2018 and 2019, indicating that appropriate N can reduce N losses (Figure 6).

### Yield and Yield-Related Traits

Nitrogen treatments significantly affected the yield traits including grains per spikes, 1000-grain weight, and grain yield of winter wheat during both years (Table 6). Among N treatments, maximum 1000-grain weight and seed yield were recorded for N225 which increased 1000-grain weight by 4.46 and 31.02% and grain yield by 29.36 and 66.78% in 2018 and 2019, respectively, compared to the control treatment (N0) (Table 6).

**TABLE 6:** Effect of nitrogen (N) application rates on number of ears per hectare, grains per spike, 1000-grain weights, and grain yield of winter wheat during 2018 and 2019.

Year	Treatment	Number of ears (10000 ha^–1^)	Grains per spikes	1000-grain weight (g)	Grain yield (kg⋅ha^–1^)
2018	N0	480.01d	30.96c	34.90d	5102.61d
	N75	503.89c	33.48b	35.61c	6006.38c
	N150	509.44bc	35.40a	35.90c	6480.00b
	N225	521.44b	34.68ab	36.86a	6600.00a
	N300	535.15a	33.84b	36.37b	6510.00b
2019	N0	473.89e	28.70d	37.97d	4104.16d
	N75	487.78d	31.97c	43.62c	5254.69c
	N150	496.11c	33.80ab	48.20ab	6500.86b
	N225	505.00b	34.57a	49.75a	6845.47a
	N300	516.67a	33.00b	47.70b	6405.68b

*N0: unfertilized control; N75: N application at 75 kg N⋅ha^–1^; N150: N application at 150 kg N⋅ha^–1^; N225: N application at 225 kg N⋅ha^–1^; N300: traditional N application rate of 300 kg N⋅ha^–1^. Different small letters show significant differences at p < 0.05.*

## Discussion

### Photosynthetic Process

Leaf area index (LAI) can be used to reflect the growth of plant population and determine the supply status of N. Photosynthetic rates directly reflect the strength of photosynthesis. The SPAD value at flowering stage indicates the relative chlorophyll content of flag leaf In our study, the SPAD value and LAI at flowering stage increased linearly with an increase of N application. Latiri-Souki et al. (1998) also demonstrated that increasing the applied amount of N improved LAI, relative chlorophyll content of flag leaf, and photosynthetic rate for better crop growth and seed yield. Net photosynthetic rate, Ci and Tr are among the important indicators of gasses exchange and photosynthetic activity of plant leaves. In this study, Pn, Ci and Tr of flag leaf were increased with increasing N and maximum values were recorded for N225. On the contrary, Gs was decreased with increasing N rate and minimum values were recorded for N225 in comparison to other treatments. Our results showed that excessive N application at 300 kg⋅ha^–1^ reduced the photosynthetic performance of winter wheat. However, N application at 225 kg⋅ha^–1^ was the most beneficial for stomatal gases exchange and photosynthesis. Some published reports also demonstrated that leaves photosynthetic rates increase with increasing N till a threshold level is reached. The number of panicles in wheat increased correspondingly under high N rate, but large population of applied N was not conducive to increase the leaf photosynthesis (Latiri-Souki et al., 1998).

### Plant N Distribution and Transportation

Nitrogen application below the recommended rate retarded the growth and developmental processes in wheat. On the other hand, higher N rates caused significant losses by adding the excess N to soil residual N pools; thereby pollutanting the environment as well (Anas et al., 2020). Nitrogen application rates significantly affected the uptake and transportation of N in winter wheat. In our work, N accumulation in different organs at flowering stage follow the trend as leaf > stem sheath > glume + cob, and N accumulation in grains was significantly increased, ranging from 66.14–173.56 kg⋅ha^–1^, accounting for 81.60–89.72% of the total N in above-ground plant parts. Also, about 53.26–71.23% of total N was transferred from vegetative organs after flowering stage, indicating that vegetative organs contribute a relatively high proportion to N accumulation in grains. Excessive N application is beneficial for accumulating N in vegetative organs at maturity stage.

### Nitrogen Efficiency

The effect of various nitrogen application rates on N efficiency of winter wheat was mainly reflected in absorption efficiency and utilization efficiency. Nitrogen absorption and utilization efficiency were different under different N rates. In this study, with exceeding N rates, nitrogen harvest index, nitrogen partial productivity and agronomic nitrogen efficiency were decreased gradually, and nitrogen absorption efficiency and N contribution rates were consistent where maximum values were recorded for N225. Similar to our findings, Wang et al. (2010) reported that increasing N rates significantly increased the wheat yield, N uptake and utilization efficiency, however, excessive rates caused a significant decline in wheat N utilization efficiency.

### Residual Nitrogen

In this study, the residual soil N was increased gradually with increase of N rate. In Guanzhong Plain area, remaining soil N after crop harvest tends to move-down after rainfall, resulting in leaching and denitrification losses. Our results showed that the nitrate-N contents are positively related to N application rates. This is consistent with previous studies where increasing N rate caused a significant increase in nitrate-N content (Wang et al., 2010). In this study, the nitrate nitrogen (NO_3_-N) was mainly concentrated in deeper soil layers (below 120 cm) and varied in different experimental years. As compared with second year of the experiment (2019), nitrate nitrogen contents (NO_3_-N) in different soil layers were lower in the first year (2018), which may be related to the effect of straw retention. Straw retention combined with N fertilizer is beneficial to improve the plant N utilization efficiency and reduce the leaching N losses. In a previous study, Oikeh et al. (2003) demonstrated that under high N application, soil residual N content kept on increasing with increasing soil depth and were maximum below 1 m soil layer. In our study, ammonium nitrogen content did not vary significantly for tested soil layer.

### Yield

Fertilizer application combined with straw retention plays an important role in sustainable agricultural production. In this study, compared with N0, grain yield was improved to some extent with exceeding N application rates with increase of 17.71, 26.99, 29.35, and 27.58% in 2018 and 28.03, 58.40, 66.79, and 56.08% in 2019 for N0, N150, N225, and N300, respectively. Straw retention combined with synthetic N fertilizer application effectively increased the wheat yield where maximum yield was recorded during second study year. N150 and N225 treatments recorded higher yield than N300. Previous published reports also showed that N application rates and grain yield showed a quadratic curve where N application increased grain yield within a certain threshold range. When N application rate exceeds the critical range, it results in lower nitrogen use efficiency and environmental pollution (Yang et al., 2017). Reduced number of grains per spike, thousand grain weight, grain yield and water and nutrient use efficiency under excessive N rate were also reported by Liu et al. (2021).

## Conclusion

N application rates significantly affected the photosynthesis, plant growth, N use efficiency and productivity of winter wheat under straw incorporation. N application at 150 and 225 kg⋅ha^–1^ was the most economical method under straw incorporation; it significantly increased the N uptake, N translocation in different plant organs including grains. Compared with N300, N150 and N225 reduced the soil nitrogen losses and in this way contributed to the sustainable agricultural development.

## Data Availability Statement

The original contributions presented in the study are included in the article/supplementary material, further inquiries can be directed to the corresponding author/s.

## Author Contributions

JW: conceptualization, methodology, investigation, formal analysis, writing – original draft, and writing – review and editing. SH: investigation, formal analysis, and writing – review and editing. XS: investigation, formal analysis, and writing – review and editing. TJ: writing – review and editing. ED: writing – review and editing. PZ: writing – review and editing. XC: conceptualization, methodology, and writing – review and editing. XR: conceptualization, methodology, and writing – review and editing. All authors contributed to the article and approved the submitted version.

## Conflict of Interest

The authors declare that the research was conducted in the absence of any commercial or financial relationships that could be construed as a potential conflict of interest.

## Publisher’s Note

All claims expressed in this article are solely those of the authors and do not necessarily represent those of their affiliated organizations, or those of the publisher, the editors and the reviewers. Any product that may be evaluated in this article, or claim that may be made by its manufacturer, is not guaranteed or endorsed by the publisher.
